# Genome Sequences of Livestock-Associated Methicillin-Resistant Staphylococcus aureus *spa* Type t899 Strains Belonging to Three Different Sequence Types (ST398, ST9, and ST4034)

**DOI:** 10.1128/MRA.01351-18

**Published:** 2019-01-10

**Authors:** Henok Ayalew Tegegne, Ivana Koláčková, Martina Florianová, Tereza Gelbíčová, Pierre Wattiau, Cécile Boland, Renáta Karpíšková

**Affiliations:** a Department of Bacteriology, Veterinary Research Institute, Brno, Czech Republic; b Faculty of Veterinary Hygiene and Ecology, University of Veterinary and Pharmaceutical Sciences, Brno, Czech Republic; c Veterinary Bacteriology, Sciensano, Brussels, Belgium; Louisiana State University

## Abstract

Livestock-associated methicillin-resistant Staphylococcus aureus (LA-MRSA) is an emerging MRSA lineage rapidly evolving in the community. In this report, we present the draft genome sequences of nine LA-MRSA strains. These strains were isolated from meat and a human nasal swab sample and belong to one unique *spa* type (t899), but to three different sequence types, ST398, ST9, and ST4034.

## Abstract (precis)

Livestock-associated methicillin-resistant Staphylococcus aureus (LA-MRSA) is an emerging MRSA lineage rapidly evolving in the community. In this report, we present the draft genome sequences of nine LA-MRSA strains.

## ANNOUNCEMENT

Livestock-associated methicillin-resistant Staphylococcus aureus (LA-MRSA) is the largest MRSA pool in humans outside the hospital setting, with livestock as a primary reservoir (1, 2). This lineage is predominantly represented by clonal complex 398 (CC398), but it also comprises other clonal complexes, including CC9 and CC5 (2 – 4).

Staphylococcus aureus protein A (*spa*) typing has a remarkable predictive power over clonal relatedness (5, 6). In most instances, a single *spa* type is strictly associated with a specific multilocus sequence type (MLST). However, some exceptions do exist, such as *spa* type t899, which is reported in multiple sequence types, namely, ST398 and ST9. In this report, we present the draft genome sequences of nine LA-MRSA strains, all belonging to *spa* type t899 but clustering in three different sequence types, ST398 (3), ST9 (5), and ST4034 (1) (Table 1).

**TABLE 1:** Livestock-associated methicillin-resistant Staphylococcus aureus *spa* type t899 isolates

Isolate ID^*a*^	Isolate source	Sample origin^*b*^	Year of isolation	ST^*c*^	No. of raw reads	Genome coverage (×)	No. of contigs	*N*_50_ (bp)^*d*^	GC content (%)^*d*^	Genome size (bp)	GenBank accession no.
SAV0154	Pork	CZ	2013	4034	772,052	64.34	178	39,112	32.91	2,781,027	QYAY00000000
SAV0987	Human	CZ	2017	398	1,109,012	92.42	196	37,830	32.79	2,922,579	QYAX00000000
SAV1035	Poultry meat	PO	2017	9	1,062,670	88.56	123	85,312	32.71	2,783,450	QYAW00000000
SAV1109	Poultry meat	PO	2017	398	699,934	58.33	303	20,030	32.82	2,857,636	QYAV00000000
SAV1146	Poultry meat	DE	2017	398	675,512	56.29	131	55,180	32.85	2,896,873	QYAU00000000
SAV1149	Poultry meat	DE	2017	9	893,060	74.42	277	21,660	32.79	2,749,354	QYAT00000000
SAV1150	Poultry meat	DE	2017	9	808,170	67.35	99	78,511	32.75	2,756,154	QYAS00000000
SAV1158	Poultry meat	DE	2017	9	1,231,020	102.58	64	135,732	32.73	2,764,912	QYAR00000000
SAV1228	Pork	CZ	2017	9	2,321,580	193.47	116	80,797	32.71	2,730,307	QYAQ00000000

*^a^*ID, identification.

*^b^*CZ, Czech Republic; DE, Germany; PO, Poland.

*^c^*ST, sequence type.

*^d^N*_50_ value and GC percentage were calculated based on contigs of ≥500 bp.

Isolates were obtained from meat samples collected in retail markets and from a nasal swab sample from a dialysis patient that was taken during hospital screening in the Czech Republic. The meat from which the samples were drawn was produced in different countries and sold in the Czech Republic. The samples were primarily enriched in buffered peptone water and cultured on Baird-Parker agar. Presumptive S. aureus colonies were transferred to blood agar and confirmed using matrix-assisted laser desorption ionization–time of flight mass spectrometry (MALDI-TOF MS) (7). All MRSA isolates were identified using PCR detection of the S. aureus-specific fragment SA442 and the *mecA* gene (8). MLST (https://cge.cbs.dtu.dk/services/MLST) (9) and *spa* typing (https://www.spaserver.ridom.de) (10) were performed prior to the whole-genome sequence run.

Total genomic DNA was extracted using a DNeasy blood and tissue kit (Qiagen, Valencia, CA) from pure culture colonies cultivated on Columbia sheep blood agar (Bio-Rad Laboratories, Temse, Belgium). Whole-genome sequencing was performed with a MiSeq sequencing platform (Illumina, San Diego, CA). Library preparation was performed with the Nextera XT DNA sample preparation kit (Illumina). The libraries were then sequenced using a 250-bp paired-end protocol (MiSeq reagent kit v.3, Illumina) according to the manufacturer’s instructions. Data analysis was performed using an in-house instance of the Galaxy workflow management system (11). Sequencing yielded a total of 9,573,010 reads with 35- to 251-bp read lengths. Raw reads were quality checked with FastQC v.0.65, and low-quality reads were trimmed using Trimmomatic v.0.36.4 (12). Subsequently, assemblies were generated using the SPAdes v.1.3.1 algorithm (13). Contigs ≥200 bp long were retained in the assembly. The genome sizes ranged from 2,730,307 to 2,922,579 bp. The average GC content and the *N*_50_ value were 32.8% and 61,574 bp, respectively. Final assemblies consisted of 64 to 303 contigs with an average coverage of 88.64× (Table 1). Annotation was carried out using the NCBI Prokaryotic Genome Annotation Pipeline (PGAP) (https://www.ncbi.nlm.nih.gov/genome/annotation_prok/) (14).

### Data availability.

The genome sequences reported here have been deposited at DDBJ/ENA/GenBank under the accession numbers QYAQ00000000 to QYAY00000000. The versions described in this paper are the first versions, QYAQ01000000 to QYAY01000000 (Table 1). Raw sequences are available under the SRA study accession number SRP161670.
